# Pandemic Effects: COVID‐19 and the Crisis of Development in the Middle East

**DOI:** 10.1111/dech.12727

**Published:** 2022-07-27

**Authors:** Adam Hanieh, Rafeef Ziadah

## Abstract

This article explores the effects of the COVID‐19 pandemic on socio‐economic development and political mobilization in the Middle East. It argues that beyond its direct public health implications, the pandemic is serving to intensify the extreme differences in wealth and power that have characterized the region for many years. The article gives an overview of the region's political economy prior to the pandemic, examining the legacies of the 2011 uprisings and the ways in which notions of ‘crisis’ were mobilized to re‐embed market‐led development models over the last decade. Within this broader context, it maps the generalized deterioration in living conditions that has occurred since mid‐2020. Following this, it discusses the pandemic's exacerbation of regional unevenness, exploring the strengthened position of more powerful states, notably Israel and the Gulf states, within the political and economic hierarchies of the Middle East. Finally, the article takes a closer look at Lebanon, Tunisia and Sudan, three countries that have been sharply hit by the pandemic, but that were also marked by substantial mass protests and political mobilization immediately prior to 2020. These countries illustrate the political complexities involved in situations where a profound socio‐economic crisis intersects with a long‐standing erosion of political hegemony.

## INTRODUCTION

The last decade has witnessed far‐reaching changes in the political, social and economic dynamics of the Middle East.^1^ As the most conflict‐affected area of the world, the region is today marked by the largest forced displacement globally since World War II (World Bank, 2017: 26). The predicament of millions of refugees and internally displaced peoples (IDPs) contrasts starkly with the extreme levels of wealth also present in the region. Indeed, by some measures, the Middle East is now the most unequal area of the world, with 31 billionaires holding the same amount of wealth as the bottom half of the entire adult population (ESCWA, 2020a: 3). These long‐standing inequalities and the region's deep‐rooted history of authoritarian rule have spurred two major waves of political contestation over the last decade: the 2011 uprisings and a more recent cycle of mobilizations that began in 2018 (Beinin and Vairel, 2013; Saab, 2020). The aspirations of these protests remain wholly unfulfilled, but they continue to be a potent reminder of the possibility of change across the region.

Amidst this conflictual and highly unstable regional order, the spread of the COVID‐19 pandemic has had profound public health effects. By January 2022, the Middle East and North Africa (MENA) had officially recorded over 30 million cases and over 370,000 deaths due to COVID‐19.^2^ However, a significant lack of testing capacity means that these numbers give a highly misleading picture of the spread of the virus. Indeed, for many countries in the region, the positivity rate has averaged between 20 and 50 per cent during various waves of the pandemic^3^ — an indication that the virus was essentially running uncontrolled throughout the population and that test numbers were substantially understating the actual number of cases. Death rates have been notably higher in poorer countries (such as Tunisia) and those with large numbers of refugees and displaced peoples (for example, Lebanon and Jordan). For the most conflict‐affected countries in the region — particularly Syria, Libya and Yemen — it is impossible to obtain an accurate picture of the real mortality rates associated with the virus (Daw, 2021).

Given this context, there are two core arguments that we advance in this article. The first is that the effects of the pandemic are unfolding across the Middle East in a differential and highly variegated manner, serving to compound and intensify the significant differences in wealth and class power that have marked the region for many decades. This trend is evident both within and between countries. As elsewhere around the world (Stevano et al., 2021), the poorest and most marginalized groups (such as women, youth, refugees and migrants) have been heavily hit by lockdowns, workplace closures and the unprecedented contraction in economic activity that has occurred over the last 18 months. At the same time, the wealthiest states in the region (especially Israel and the six countries of the Gulf Cooperation Council ‒ GCC^4^) appear to be emerging from the pandemic in a strengthened position, a trend that will likely be reinforced by considerable divergences in the pace of vaccine roll‐out. By mapping these polarizing effects of the pandemic, we aim to move away from a widespread view of COVID‐19 as a straightforward public health emergency to one in which the pandemic is seen as a catalyst for broader transformation of the region's highly uneven and crisis‐prone capitalist development.

Framed by this broader socio‐economic trajectory of the region, the second core argument of this article examines the political dimensions of the pandemic. Here, we argue that the pandemic is exacerbating a longer‐term erosion in the political legitimacy of ruling classes, which has become increasingly acute over the last decade (Hinnebusch, 2018). Across many countries in the Middle East, established political leaderships have repeatedly shown themselves incapable of addressing the manifold dimensions of capitalist crisis, or the severe deterioration in living conditions that have marked the neoliberal period (Abdelrahman, 2014; De Smet, 2021; Gherib, 2021). As elsewhere, the state acts as a core lever of accumulation for the largest capitalist groups while ignoring wider social grievances, thereby undermining its ability to consolidate a broader legitimacy. For some countries, the lack of any durable political hegemony is manifest in repeated cycles of popular protest and social mobilization, as well as highly volatile and unstable government coalitions and ruling alliances. Paralleling global trends (Bruff, 2014; Fabry and Sandbeck, 2019), there has been a further hardening of authoritarian forms to deal with weakening state legitimacy, with modes of governance increasingly combining market‐led development models and heightened levels of surveillance, direct repression and coercive violence (Josua and Edel, 2021; Ziadah, 2021). In the wake of the unprecedented public health emergency and economic calamity induced by the pandemic, such trends have intensified.

These interlinked dimensions of the region's development — socio‐economic polarization and the weakness of political hegemony — help to clarify different ways of understanding the notion of *crisis* in relation to the pandemic. On one side, as various authors have noted in other geographical settings, crises ultimately represent moments of extreme malleability and fluidity; they are thus frequently seized upon by capitalist states and powerful actors as opportunities to restructure and push forward change in ways that were previously foreclosed (Cypher, 1989; Huws, 2012; Oguz, 2013). There are numerous historical examples of this in the Middle East, from the Volcker‐induced debt crisis of the 1980s, which propelled most Arab countries along the path of structural adjustment, to the 1990s sanctions and 2003 US/UK‐led war against Iraq, which saw the wholesale restructuring of Iraq's political economy (Dodge, 2017). Since the 2011 uprisings, however, this ‘crisis as opportunity’ motif has principally involved an effort by leading International Financial Institutions (IFIs), to re‐articulate markets as a *solution* to crisis. Organizations such as the World Bank and International Monetary Fund (IMF) have played a prominent role in shaping this narrative, utilizing the various political and economic shocks that emerged in the wake of 2011 as a means of recuperating and deepening market‐led development models (Hanieh, 2015). In many respects, the current conjuncture presents a similar moment of opportunity.

Nonetheless, the articulation of crises — both in terms of how they are identified and defined, and how they are potentially resolved — always involves contestation between different social forces. As such, the eventual outcome of this particular moment of crisis is by no means inevitable. Mirroring experiences in other areas of the world, COVID‐19 first appeared in the Middle East at a time of high levels of popular protest and widespread street mobilizations across numerous countries (notably Algeria, Iraq, Lebanon, Morocco, Sudan and Tunisia). These mobilizations presented a sharp challenge to the existing political order, combining a rejection of authoritarian and sectarian patterns of rule with the need to reverse the region's massive social and economic inequalities (Saab, 2020). Emergency measures enacted in the wake of the pandemic initially dampened these protest movements, but they were to re‐emerge across several countries in 2021. Despite a regional re‐entrenchment of authoritarianism, the political forms and strategic legacies of these earlier struggles remain highly pertinent to the current moment. All this means that the trajectories of social contestation around the crisis of development in the region remain very much open‐ended.

In illustrating these different aspects of the pandemic and crisis in the region, we focus below on the Arabic‐speaking countries of the Middle East, as well as Israel in the context of its occupation of the West Bank and Gaza Strip. The article proceeds through four main sections. We begin with a brief overview of the region's development prior to the pandemic, examining the legacies of the 2011 uprisings and how the notion of crisis was mobilized by IFIs and other powerful actors in the decade following these mass protests. The second section of the article concentrates on the immediate socio‐economic impact of the pandemic, mapping the generalized deterioration in living conditions that has occurred over the last 18 months. We then turn to the pandemic's consequences for the dynamics of the regional scale, exploring the strengthened position of more powerful states, notably Israel and the Gulf states, within the broader hierarchies of the Middle East (and the ways that these states have partially displaced the pandemic's effects onto non‐citizen workforces). Finally, we take a closer look at three countries — Lebanon, Tunisia and Sudan — that have been sharply impacted by the pandemic, but that were also marked by substantial mass protests and political mobilization prior to 2020. These countries illustrate the political complexities involved in situations where a profound socio‐economic crisis intersects with a long‐standing erosion of political hegemony.

## ‘CRISIS AS OPPORTUNITY’: PRE‐PANDEMIC DEVELOPMENT TRAJECTORES AND THE 2011 UPRISINGS

The political economy of the contemporary Middle East has been profoundly shaped by the economic shifts that began in the 1980s under the aegis of leading international financial institutions. In line with conditionality agreements established as part of structural adjustment packages, most governments in the region moved through subsequent decades to reorient their economies away from the state‐led development models of the post‐war period (Beinin, 2001; Bogaert, 2013). The policies adopted differed little from those found elsewhere around the globe: the prioritization of private sector growth, fiscal austerity and cuts to subsidies, trade liberalization, opening‐up to foreign capital inflows, export‐oriented and import‐dependent agriculture, and the deregulation of markets (including labour) (Hanieh, 2013). There was no essential contradiction between the roll‐out of these economic policies and a deepening turn towards political authoritarianism — indeed, the opening‐up of markets was driven by autocratic regimes supported by Western governments and IFIs (Abdelrahman, 2012).

Through the 1990s and 2000s, these economic shifts generated large and persistent disparities in the ownership and control of wealth, access to resources and markets, and the exercise of political power. On one side, a tiny layer of the region's population benefited from the economic transformation, with privatization and new market opportunities presenting lucrative openings for well‐connected business groups, state and military elites. Simultaneously, however, the rest of the region's population experienced social and economic conditions that persistently ranked among the worst in the world (Achcar, 2020; Bayat, 2013; Heydarian, 2014). Levels of poverty, unemployment and informal work remained very high, most particularly among youth and women. Hardest hit were poorer populations, especially those in rural areas and informal urban settlements, where many lived and worked in precarious conditions without access to social protection (Ayeb and Bush, 2019; Fawaz, 2009; Ismail, 1996). Alongside these widening social inequalities at the national scale, new economic and political hierarchies also developed at the regional level. Central to this was the growing dominance of the six GCC states in the region's political economy (Hanieh, 2018).

These uneven outcomes of capitalist development mean that external shocks — whether arising from war, foreign intervention or economic recession — tend to have a heterogeneous impact across the region, often amplifying and intensifying differences in power between states, classes and social groups. A good illustration of this was the impact of the global economic crisis of 2008‒09. Most non‐oil exporting states in the region were hard hit by this global crisis, which not only caused a sharp drop in exports, overseas remittances and income from tourism and foreign direct investment, but also occurred in an environment of high world food and energy prices. The overall effect of this was felt most by poorer classes and weaker countries. In contrast, in the Gulf states the direct impact of the crisis was cushioned using financial surpluses earned during the years of high oil prices that preceded the global collapse. Moreover, with a majority of its workforce composed of temporary migrant workers, the Gulf was able to displace the main burden of economic contraction and unemployment onto neighbouring countries — the hiring of new workers slowed, and existing workers were sent home as projects were cancelled (Hanieh, 2013). These divergent regional trajectories meant that the Gulf states emerged from the 2008 crisis in a strengthened position: the dominance of Gulf capitalism at the regional scale was accentuated, whilst neighbouring countries experienced a series of acute social and economic crises.

These polarized outcomes of national and regional development drove a series of mass uprisings that first emerged in Tunisia in December 2010 and spread rapidly throughout the entire region. The first phase of these protests through 2011 saw the overthrow of the Ben Ali regime in Tunisia and the Mubarak regime in Egypt. Governments in Syria, Bahrain, Jordan, Algeria, Oman, Morocco, Yemen and Libya also experienced uprisings and protests that expressed opposition to autocratic rulers and deteriorating living conditions (Beinin and Vairel, 2013). In this fashion, the uprisings reflected both deep‐seated anger at the uneven results of market‐led development models, as well as a desire to overturn the authoritarian political structures that drove the implementation of these neoliberal policies (Abdelrahman, 2012; Bogaert, 2013; Hanieh, 2013). Most significantly, the uprisings also highlighted the weakness of ruling class hegemony, particularly in those states approaching an imminent generational transition in authoritarian rule, such as Egypt and Tunisia (De Smet, 2021; Gherib, 2021). Faced with worsened living standards and various symptoms of social decay, the region's ruling classes and political elites were seen as incapable of managing the state of permanent crisis.

The initial response to these uprisings witnessed a variety of different forms of foreign intervention, with Western powers endeavouring to steer them in directions that did not disrupt their long‐standing strategic interests in the region (Allinson, 2019; Hinnebusch, 2018). An important element to this was an attempt to ensure the continuity of market‐led development strategies, and here the discursive reframing of the region's ‘crisis of development’ (and its possible resolution) was key. According to the arguments presented by institutions such as the World Bank and IMF, the principal reason for this crisis lay in the stifling effect of authoritarianism on capitalist markets and private‐sector growth; in this manner, poverty, unemployment, inequality and myriad other problems were portrayed as outcomes of state dominance that could be cured by further and more extensive market reforms (Hanieh, 2013, 2015). The uprisings were thus depicted as an expression of the region's yearning for ‘democracy’ on the one hand, and ‘open’ economic markets on the other. In this vein, then‐US President Obama would argue in May 2011 that the region needed ‘a model in which protectionism gives way to openness, the reins of commerce pass from the few to the many, and the economy generates jobs for the young. America's support for democracy will therefore be based on ensuring financial stability, promoting reform, and integrating competitive markets with each other and the global economy’ (White House, 2011).

Driven by this logic, the various regional crises that followed 2011 were to be recast by IFIs and other development actors as openings to consolidate and re‐embed market‐led economic models in the region. As the European Investment Bank (EIB) commented in early 2011, shortly after the overthrow of the Mubarak regime, the uprisings had ‘increased uncertainty and instability’ but such ‘moments of political change … also represent[ed] an opportunity to reinforce or improve already existing institutional frameworks’ (EIB, 2011: 12). Despite widespread popular protests against IFI conditionalities, the IMF and World Bank were to negotiate new loan packages in Tunisia, Morocco and Egypt (Hanieh, 2015). And in a highly symbolic act, the European Bank for Reconstruction and Development (EBRD) — an institution established to steer Europe through its own earlier period of crisis — would enter the region for the first time in history, with its President noting that ‘The EBRD was created in 1991 to promote democracy and [a] market economy [in Eastern Europe], and the historic developments in Egypt strike a deep chord at this bank’ (Reuters, 2011). Even the devastating wave of human displacement resulting from the wars in Syria, Yemen and Libya would be leveraged to promote further economic liberalization; a major international conference convened in 2016 would go so far as to urge donors ‘to turn the Syrian refugee crisis into a development opportunity’ through providing financial aid for programmes that ‘expand investment, promote exports and public‒private partnerships’ (OECD, 2016).

These dynamics confirm the conclusions of a wider set of scholarly literature, which identifies crisis and conflict as opportune moments for capitalist restructuring, where new policies can be introduced that reshape expectations, change social norms and shift patterns of capital accumulation (Cramer, 2006; Turner, 2017). Yet it is important to place this alongside the high levels of political contestation and social mobilization that have repeatedly enveloped the Middle East over the last decade, as well as the inability of ruling regimes to substantively address the deteriorating conditions experienced by most of the region's population. These contrasting dimensions of capitalist development — marked by unevenness, polarization and contestation — are crucial to understanding the impact of the COVID‐19 pandemic as a catalyst for social and economic transformation in the Middle East.

## SOCIAL AND ECONOMIC EFFECTS OF THE COVID‐19 PANDEMIC

The long‐standing crisis facing the Middle East as it entered 2020 was evident in a range of social and economic indicators. Just prior to the pandemic, the region's unemployment rate had reached the highest of anywhere in the world, standing at double the world average (ESCWA, 2020b: 106). Young people and women were among the most marginalized, with regional female unemployment averaging 20 per cent (and almost double that figure for young women); of the 14 worst countries in the world for female employment, 12 were located in the Middle East (CRS, 2020: 15). The rate of labour force participation for women (and young women) was also the lowest in the world — only 27 per cent of the region's youth (15‒24 years), and 19.6 per cent of women, were in work or actively seeking work just before the pandemic.^5^ For those with a job, a large majority were employed in informal and precarious work. In 2019, informal employment ranged from 45 per cent of total non‐agricultural employment to more than 75 per cent in some countries (ESCWA, 2020c: 18). If agricultural work is included, some 61.8 per cent of the region's women workers and 85.1 per cent of its youth were in informal work on the eve of the pandemic (ILO, 2018: 25).

Nonetheless, as the previous decades had amply illustrated, this was not a story of universal decline. A small layer of the region's population enjoyed considerable increases in wealth, including some of the Gulf monarchies whose per capita GDP rates ranked among the highest in the world. Buoyed by a near decade of high oil prices, the gap between the Gulf states and other neighbouring Arab countries widened substantially in the period following the Arab uprisings. Indeed, according to one widely cited study, the Middle East was the most unequal region in the world on the eve of the pandemic, with the richest 10 per cent of income earners capturing 64 per cent of total income — compared to 37 per cent in Western Europe, 47 per cent in the United States and 55 per cent in Brazil (Alvaredo et al., 2019). The figures were even starker for the ultra‐rich population of the region: the income share of the top 1 per cent stood at about 30 per cent in the Middle East, compared to 12 per cent in Western Europe, 20 per cent in the US, 28 per cent in Brazil, 18 per cent in South Africa, 14 per cent in China and 21 per cent in India (ibid.).^6^

Given these divergences, and echoing some of the historical experiences surveyed above, the pandemic has served to accentuate inequalities across the region, with its effects falling disproportionally on the poor and most marginalized, particularly women, youth, migrants and refugees. A key illustration of this can be seen in the pandemic's impact on labour markets and employment. In the wake of lockdowns and workplace closures — and compounded by a contraction in economic activity — both the quantity of jobs (levels of unemployment and underemployment) and quality of jobs (wages, income and social protection) have worsened considerably (ILO, 2020: 5). As Table 1 illustrates, labour force participation rates and unemployment deteriorated significantly over 2020. Although numbers were predicted to have recovered slightly by the end of 2021, the overall situation remained much worse than 2019. Women have seen their labour force participation levels drop faster than men, and 22 per cent of women in the labour market are now estimated to be unemployed. Youth unemployment rates have also increased, reaching around 32 per cent in Morocco, 36.5 per cent in Tunisia, and 55 per cent in Jordan in the fourth quarter of 2020 (between 3 to 10 percentage points above 2019 levels) (IMF, 2021a: 5). The ILO estimates that the Arab countries of the Middle East lost 9.1 per cent of their working hours in 2020 and another 5.4 per cent in 2021 — higher than the global averages of 8.6 per cent and 3.9 per cent for 2020 and 2021 respectively. These losses are equivalent to more than 8.8 million full‐time equivalent jobs.^7^

**Table 1:** Labour Force Participation (LFP) and Unemployment (UE) Rates during and preceding the Pandemic (aged 15+), MENA Region

	LFP	LFP	LFP	UE	UE	UE
	2019	2020	2021	2019	2020	2021
**Male**	71.6	69.8	70	7.4	8.8	8.7
**Female**	19.4	18.4	18.6	19.4	21.5	22
**Total**	46.5	45.1	45.3	9.8	11.3	11.3

*Note*: Figures for 2021 are estimates.

*Source*: ILOSTAT database, figures for MENA (www.ilostat.ilo.org/data/; accessed 3 September 2021).

These plummeting levels of employment have had a major impact on poverty levels. It is predicted that nearly one‐third of the population of Arab middle‐income and least developed countries will fall below the headcount poverty rate as a result of the pandemic, bringing the number of poor in these countries to 115 million people (AFSD, 2021: 3). The number of people in extreme poverty is expected to increase by a further 9 million people in the Arab countries of the Middle East, excluding the GCC. The pandemic has also highlighted the vulnerabilities of the ‘working poor’ — working individuals whose incomes are not sufficient to lift them and their families out of poverty. According to the ILO, the numbers of working poor rose by 20 per cent in 2020, a growth of 3.6 million people (ILO, 2021b: 44, 63). A large proportion of the working poor are informal sector workers who lack job security, adequate savings and access to paid leave — indeed, the ILO estimates that 89 per cent of all informal workers in the Middle East were affected by workplace closures and lockdowns during the pandemic (ILO, 2020: 4).

These labour market and poverty trends are also highly gendered, impacting women much more than men. Already ranked at the bottom of the world in terms of labour force participation, women have been driven out of the labour market at a faster rate than men (see Table 1 above). Moreover, the proportion of unpaid work performed by women in the Middle East was greater than any other region in the world prior to the pandemic (ESCWA and UN Women, 2020: 2), and this labour has increased following the closure of schools, childcare and other care facilities. In many countries in the region, women make up a majority of health care and social support workers (in Egypt, for example, women outnumber men 10:1 among nursing staff), and thus have faced not only a double burden of longer working shifts and unpaid care work at home, but also a disproportionate risk of exposure to infection itself. Women's access to reliable information about the virus (and other health issues) is also hampered by a lack of internet connectivity — nearly half of the female population in Arab countries do not have connection to the internet or access to a mobile phone (ESCWA and UN Women, 2020: 4).

The region's 26 million refugees and internally displaced people have been particularly vulnerable to the social and economic consequences of the pandemic. While there are major gaps in COVID‐related data for this population group (Wehbe et al., 2021), refugees and IDPs tend to be economically precarious and located in areas with high population density and poor water, sanitation and hygiene infrastructure. These factors have made it more difficult to self‐isolate and enact necessary public health measures during the pandemic. Conflict has also destroyed health infrastructure and interrupted critical care, placing further pressures on national health systems (UN, 2020: 4). For households reliant on humanitarian aid (an estimated 55 million people in the region), the pandemic's impact on supply chains has threatened to disrupt the provision of food, water and sanitation, medical supplies and health services. Countries affected by conflict and displacement have seen a major rise in poverty levels, which further increases the precarious situation of refugees and IDPs. ESCWA estimates poverty rates in Lebanon and Jordan, for example, where a large proportion of the region's refugee population live, at 23.1 per cent and 57.9 per cent respectively in 2020 (ESCWA, 2021: 15). In Syria and Yemen, more than 80 per cent of the population now live in poverty (ibid.).

Another route through which the pandemic has exacerbated inequalities and further eroded the living conditions of the poor is through its effect on food security and agriculture. Following liberalization of the agricultural sector and the move towards export‐oriented production over the last few decades, the region has become heavily dependent upon food imports (Ayeb and Bush, 2019). Rising food prices through 2020 and 2021 — partly driven by the impact of the pandemic on trade and global supply chains — has meant a large growth in the cost of imported food. Compounded by the overall drop in household incomes, this has generated greater levels of food insecurity in the region, as well as more reliance of many families on humanitarian food aid. Agricultural workers and small‐scale farmers have also been hard hit by the shutdown of farms and closure of traditional food markets (FAO, 2020). The livelihoods of rural women have particularly suffered; women make up a majority of the agricultural workforce in most MENA countries, and almost all of this labour is informal (Abdelali‐Martini and Dey de Pryck, 2015). In areas where agricultural work continued throughout the pandemic, this has raised the risk of infection for women. In Morocco, for example, the largest outbreak of the virus during the initial months of the pandemic occurred among hundreds of women strawberry pickers working for Spanish‐owned firms in the Kenitra region (Whitaker, 2020).

These various trends highlight the deep social and economic consequences of the region's turn towards market‐led development strategies over recent decades. To a significant degree, the differential effects of the pandemic on various communities, classes and social groups were channelled through the outcomes of the preceding years of economic reform. The privatization of social services, for example, undermined the capacity of health and education systems to mitigate the impact of the virus on the poorest communities. The liberalization of the agriculture sector — and especially the region's high dependency on food imports — increased the level of food insecurity across many countries (and the difficulties faced by agricultural workers and their families). The deregulation of labour markets made those in precarious and informal work much more susceptible to both the economic *and* health effects of the pandemic. As a result, the pandemic has served as a catalyst, magnifying and amplifying the fundamental characteristics of capitalist development in the region.

## REGIONAL DIVERGENCES: ISRAEL AND THE GCC STATES

These aggregate regional trends confirm the highly destructive impact of COVID‐19 on social and economic conditions in the region. However, in examining the events of the last 18 months, it is essential to also foreground the widening regional hierarchies that have opened up through the course of the pandemic. A small subset of countries, notably Israel and the Gulf states, were able to mitigate the pandemic's immediate impact much more successfully than the rest of the region. This was partly due to the greater financial capacities of these states, which allowed them to substantially raise government expenditure in the initial phases of the pandemic while many neighbouring countries were unable to do so.^8^ As a result, Israel and the GCC did not suffer the same deterioration in labour market conditions, employment and poverty rates as was evident in other parts of the region (even though initial infection rates in Israel and the GCC were much higher). According to recent IMF estimates of key financial indicators, this divergence between Israel and the GCC, on one side, and the rest of the Middle East, on the other, is expected to grow over 2022 (see Table 2).

**Table 2:** Estimates of GDP Growth, Current Account Balances and General Government Gross Debt 2020‒22

	Real GDP Growth (2020)	Current Account Balance (% of GDP 2020)	Current Account Balance (% of GDP 2021)	Current Account Balance (% of GDP 2022)	General Government Gross Debt (% of GDP 2020)	General Government Gross Debt (% of GDP 2021)	General Government Gross Debt (% of GDP (2022)
**GCC**	‒4.8	‒1.3	4.2	3.8	41.0	38.3	39.6
**Israel**	‒2.2	5.4	4.5	3.8	72.0	73.2	73.2
**Iraq**	‒10.9	‒14.8	0.0	‒0.6	81.2	69.7	73.3
**Algeria**	‒6.0	‒10.5	‒7.7	‒8.7	53.1	63.3	73.9
**Lebanon**	‒25.0	‒14.3	n/a	n/a	150.4	491.8	n/a
**Libya**	‒59.7	‒11.4	3.9	0.2	n/a	n/a	n/a
**Egypt**	3.6	‒3.1	‒3.9	‒4.0	89.8	91.4	89.5
**Tunisia**	‒8.8	‒6.8	‒9.5	‒9.4	87.6	91.2	93.9
**Jordan**	‒2.0	‒8.1	‒8.3	‒4.0	88.5	91.2	91.0
**Morocco**	‒7.0	‒2.2	‒3.8	‒4.0	76.1	77.1	77.4
**Sudan**	‒3.6	‒17.5	‒11.2	‒13.5	262.5	211.7	185.9
**West Bank & Gaza Strip**	‒11.0	‒9.0	‒10.5	‒10.8	47.3	47.9	44.3
**Yemen**	‒5.0	‒2.4	‒8.5	‒7.8	83.2	73.0	67.9

*Source*: IMF (2021a, Tables 15 and 20; 2021b, Tables A7 and A15).

Alongside their greater financial capacities, a further important dimension to Israel and the GCC's pandemic response was the displacement of many of the negative economic effects of COVID‐19 onto non‐citizen workforces. In the case of the Gulf, this has involved intensified attacks on the rights of the temporary migrant workers who make up more than half the total labour force in the GCC, and perform most work in sectors such as construction, domestic work, agriculture and food production, retail, health and transport. With residency rights tied to employment contracts, hundreds of thousands of migrant workers were forced to return home during the first few months of the pandemic, as business closures and lockdowns spread throughout the Gulf.^9^ As a result, the Gulf's migration corridors served as a transmission belt for economic shock to the rest of the Middle East and Asia; the burdens of employment collapse and economic shutdown were carried principally by poorer households in surrounding regions, not in the Gulf itself.^10^

Migrant workers who remained in the Gulf also experienced considerable hardship during the pandemic. Human rights organizations have documented widespread cases of migrants who have had their wages withheld or cut, or who were forced to continue working in unsafe, overcrowded and unsanitary conditions throughout lockdown (Equidem, 2020). Government furlough or wage support schemes were directed only to citizen employees, and some of the region's largest and most profitable companies refused to pay migrants who were employed through sub‐contractors.^11^ Many migrants were stranded in their countries of employment due to a lack of operating flights or an inability to obtain the necessary COVID‐19 tests, and were denied access to food, shelter and other services (ESCWA, 2020d: 2). While there is no accurate breakdown of COVID‐19 infections in the Gulf based on citizenship status, migrant workers are also widely understood to make up a disproportionate share of infections (Wright, 2020).

The Gulf's displacement of the costs of the pandemic onto poorer populations can also be seen in vaccination policies. During the first half of 2021, all Gulf states began to mandate vaccinations for incoming migrant workers to the Gulf (including returning workers), otherwise they would be required to undergo quarantine at their own expense, at a price that was many times that of a typical workers’ monthly salary (Farooq, 2021). In doing so, the financial and logistical burden of vaccinating the Gulf's working classes was shifted onto poorer neighbouring countries that were dependent on remittances from the Gulf. This further exacerbated inequalities in migrant‐sending countries, forcing them to prioritize the vaccination of healthy and relatively young populations (migrants due to depart for the Gulf are generally under the age of 40) instead of more vulnerable and older people. Moreover, for some Gulf countries (e.g. Saudi Arabia and Kuwait), the only approved vaccinations were Western‐developed vaccines that were not widely available in key migrant‐sending countries such as Pakistan, Bangladesh and Nepal.^12^

Israel's experience over the last 18 months also illustrates how some of the immediate economic shock of the pandemic was displaced onto a mobile, cross‐border workforce — in this case, Palestinian workers from the West Bank. Since the beginning of Israel's occupation of the West Bank and Gaza Strip in 1967, Palestinians have formed a low‐wage and highly flexible labour force for several key sectors of the Israeli economy (Farsakh, 2005). Just prior to the pandemic, around one in eight Palestinian workers (13.2 per cent in 2019) were employed in Israel or in Israeli settlements in the West Bank. These workers are overwhelmingly male and concentrated in Israel's construction industry (64 per cent), manufacturing and quarries (13 per cent), or hotels and restaurants (11 per cent) (PCBS, 2019: 75). The infrastructure of Israel's military occupation in the West Bank — including checkpoints, movement restrictions and a complex permit system — serves to lower wages and magnify the precarity and vulnerability of this cross‐border workforce. The location of these workers in a geographically distinct but economically dependent territory — governed administratively by the Palestinian Authority — creates a captive workforce whose supply can be turned on and off depending on the requirements of Israeli capitalism.

With the onset of the pandemic, the closure of checkpoints in the West Bank presented major problems for the Israeli economy due to the inability of thousands of Palestinians to access work in Israel. In the construction sector, for example, the Israeli Builders’ Association initially predicted monthly losses of up to US$ 1.4 billion, as well as knock‐on effects that would disrupt the employment of over 125,000 Israelis (Gazit, 2020). Palestinian workers from the West Bank alone are estimated to generate 66 per cent of the sector's US$ 24 billion annual contribution to Israel's GDP (ibid.). In response to this potential crisis, the Israeli government agreed a deal with the Palestinian Authority on 17 March 2020 to permit the entry of workers into Israel for the construction, health and agricultural sectors. These workers were required to stay continuously in Israel for one month and were supposed to be provided with accommodation, on‐site social distancing and protective equipment. Many workers, however, did not receive these services, and were instead forced to sleep in large groups on construction sites or in warehouses lacking adequate hygiene facilities (Shezaf, 2020). Employers also withheld the ID cards of Palestinians in order to prevent them from leaving their places of work — an act that had previously been described by the Israeli Ministry of Justice as a marker of forced labour (ITUC, 2021: 14). Through these means, Israel managed to ensure the continued operation of major economic sectors throughout the first phases of the pandemic, despite the risks this presented for Palestinian workers and their families (ILO, 2021a).^13^

The pandemic has also seen the development of new digital tools in both Israel and the Gulf, which have been used to expand surveillance and control of non‐citizen workforces. In April 2020, Palestinian workers from the West Bank were forced to use a mobile application developed by the Israeli Ministry of Defence to apply for permits to cross into Israel for work (7amleh, 2020). This app, called *al‐munasiq* (‘the coordinator’), gave the Israeli military access to the user's phone, including location information, incoming and outgoing calls, emails, microphone and camera. In order to install the app, users were required to consent to their information being used ‘for any purpose, including for security purposes’ and to allow the Israeli military to ‘store the information … provided to us in our databases based on our considerations’ (Hasson, 2020). More than 50,000 workers had downloaded the app before a court ruling ordered the military to change the terms of installation (Who Profits, 2020). Likewise, the Gulf states have developed invasive COVID‐tracking apps that enable real‐time surveillance of resident populations. In Bahrain and Kuwait, for example, contact tracing apps capture location data through GPS, which is uploaded to a central database and tracks user movements at all times. These data are directly linked to individual identities and paired with a Bluetooth‐enabled bracelet used to enforce quarantine. In the wake of these measures, Amnesty International described the Bahraini and Kuwaiti apps as ‘highly invasive surveillance tools which go far beyond what is justified in efforts to tackle COVID‐19’ and among the ‘most dangerous’ for privacy in the world (Amnesty International, 2020).^14^

Moving forward, the regional divergences discussed in this section are likely to widen as a consequence of major inequalities in the distribution of vaccines. By January 2022, Israel and the Gulf states (with the exception of Oman) had fully vaccinated more than 65 per cent of their populations. At the same time, 10 countries in the region, including the most conflict‐affected states, recorded less than 40 per cent of their population fully vaccinated (with six of these at less than 15 per cent vaccination coverage).^15^ The IMF believes that most countries in the region will not have reached significant levels of vaccination before mid‐2022 or 2023 at the earliest (IMF, 2021a: 2). These vastly contrasting vaccination rates will determine the severity of future waves of infection and the ability to combat more infectious COVID‐19 variants such as Omicron. The slow pace of vaccination across most of the region will also place more pressure on government budgets and medical infrastructures in countries that are already least well‐equipped to combat the virus. Vaccination roll‐out will thus play a major role in shaping economic trajectories over the coming years: the divergent paths out of the crisis foreshadow future regional inequalities.

## PANDEMIC POLITICS: REPRESSION, LEGITIMACY AND SOCIAL PROTEST

These manifold effects of the pandemic on socio‐economic conditions in the region carry important implications for understanding the potential political impact of COVID‐19. As noted, in the years preceding the pandemic, the Middle East had seen a resurgence of social mobilization that echoed many of the demands of the 2011 uprisings. This cycle of protest — which swept Algeria, Iraq, Lebanon, Morocco, Sudan and Tunisia from 2018 — explicitly linked both the political and economic dimensions of capitalist development in the region (Saab, 2020). While the pandemic initially served to short‐circuit these protests, the events of the last 18 months have nonetheless continued to accentuate the wider erosion of political legitimacy facing many governments in the region. Catastrophic failures in addressing the pandemic and its social effects have exposed the weakness of state capacity in sectors that have been undermined by decades of market‐led reform, notably health, education and social protection. In this context, popular mobilizations began to revive from late 2020 onwards, further highlighting the brittle nature of ruling class political hegemony across the region.

Faced by this widespread legitimacy crisis, political elites have increasingly come to rely upon authoritarian forms of rule to secure their position. This entrenchment of authoritarianism was already evident in the years that followed the 2011 uprisings (Bellin, 2012; Heydemann and Leenders, 2014; Lynch, 2012), in which the tropes of ‘security’ and ‘public safety’ came to define an official political discourse mobilized around a notion of permanent emergency (Abdelrahman, 2017). In the wake of the pandemic, however, not only has state‐backed repression continued to be directed against political opposition and popular mobilization, but the place of the military and security forces has itself undergone a crucial shift, becoming ever‐more centrally located within the decision‐making and coordinative functions of the state apparatus (including at the economic level). Ideologically, this is reflected in the increasing projection of the military as the ‘guarantor of the nation’ — as well as the criminalization of any popular sentiments against these repressive forces (often framed around notions of ‘morality’).

These dynamics are clearly illustrated in the case of Lebanon, which is experiencing what the World Bank claims may be within the top three of the most severe economic collapses anywhere in the world since the mid‐19th century (World Bank, 2021a). Just prior to the pandemic, the country witnessed massive nationwide mobilizations against the deteriorating economic situation, failing infrastructure and rampant government corruption. Dubbed the ‘October Revolution’, these protests brought thousands onto the street in mass gatherings across all parts of Lebanon (Bou Khater and Majed, 2020). Notably, the slogans raised in these 2019 demonstrations criticized the sect‐based power‐sharing agreement that is the basis of the country's political system, and which has long been a lucrative source of wealth for well‐connected individuals linked to the state and different sectarian groups (Salloukh et al., 2015). Protestors understood these sect‐based patronage networks as a crucial factor behind widespread state corruption, as well as the endemic failure of essential infrastructure and services, especially electricity and waste collection (Abu‐Rish, 2014; Verdeil, 2018).

In the shadow of these mass protests and a thoroughgoing challenge to the character of the political system, the first cases of COVID‐19 were recorded in Lebanon on 21 February 2020. Cases remained relatively low in the initial months of the pandemic (in part due to a lack of testing capacity), but a large wave of infection from the second half of 2020 through to June 2021 saw the number of COVID‐19‐related deaths reach the highest of anywhere in the region (after which deaths in Tunisia began to surpass those in Lebanon). The impact of this public health emergency was amplified by a range of other major shocks, including the August 2020 Beirut Port Blast (which killed more than 200 people, injured more than 6,000, and left 300,000 homeless) and a banking crisis that saw the Lebanese lira lose more than 90 per cent of its value. As a largely service‐based economy, the 25 per cent plunge in GDP over 2020 severely hit the informal sector, especially workers in retail, tourism and transport. The price of essential consumer goods, including food and fuel, skyrocketed — with the World Food Programme recording a 340 per cent increase in the price of the basic food basket in May 2021.^16^

Faced with these multiple and intersecting forms of crisis in Lebanon, political authority has become ever‐more fractious and volatile throughout the pandemic.^17^ Both the government and the leading sectarian parties have attempted to address the collapse in political legitimacy by providing top‐down public health services as a means to reassert influence in particular sub‐national and urban geographies (Harb et al., 2021).^18^ Nonetheless, the legacies of the 2019 demonstrations continue to be strongly felt, with protests and strikes re‐emerging through 2021. The Lebanese military and security forces have intensified repression against demonstrations and street mobilizations, utilizing live ammunition, rubber‐coated metal bullets, tear gas and mass arrests. Outside of direct repression of protest, there has been a notable rise in the use of defamation laws against activists and journalists (which can carry a prison term of up to three years in Lebanon), the monitoring of online social media posts by the country's ‘Cybercrimes Bureau’, and the use of anti‐terror regulations and military courts to detain and prosecute protestors on charges of violence against security forces or ‘insulting’ the army (Amnesty International, 2021).

The inability of existing political structures to address the wider public emergencies spawned by the pandemic is further demonstrated in the case of Tunisia. Often hailed as the sole success story of the ‘Arab Spring’, Tunisia's political system has nonetheless been marked by ongoing instability, weak coalitions, frequent changeover of prime ministers, and low voter turnout, especially among youth (Powers, 2019). Successive coalitions between the Muslim Brotherhood‐affiliated Ennahda and Nidaa Tounnes (initiated by figures associated with the former Ben Ali regime) have largely adhered to the neoliberal conditionalities imposed by IFIs and have failed to address the deteriorating social conditions (Erol, 2020). The pandemic pushed this volatile political situation to the brink, with the Health Ministry reporting in early July 2021 that the public hospital system had collapsed under the pressure of hospitalizations. The country continues to be ravaged by very high levels of COVID‐19‐related deaths — by far the most in the region according to official figures — as well as a severe economic recession (see Table 2 above).

Nonetheless, as in Lebanon, a new wave of popular mobilizations had emerged in Tunisia prior to the pandemic. These included the *manich msamah* [I do not forgive] protests against an immunity bill for members of the former Ben Ali regime, the *fech nestenew* [What are we waiting for] protests against a 2017 IMF‐imposed finance bill, mobilizations against an EU Free Trade Agreement, farmer and worker actions to reclaim land and factories, and continued protests against police brutality (Riahi and Hamouchene, 2020; Zemni, 2021). These struggles have continued despite the pandemic, and have been met with increased repression by the country's security forces and police. Indeed, during a large wave of countrywide protests against the economic conditions in early 2021, over 1,500 activists were arrested in demonstrations and night‐time house raids by Tunisian security forces. Much like Lebanon, those arrested have been charged with crimes such as ‘insulting a police officer’, ‘committing an immoral act in public’ and ‘offending good morals and public indecency’ (HRW, 2021). Given this conjuncture — a fractious ruling class unable to establish a stable political order, and high levels of popular mobilization outside of the parliamentary system — the country's president, Kais Saied, suspended parliament in July 2021 and evoked Article 80 of the Constitution, which gave him exceptional powers ‘in the event of imminent danger’. This increasingly Bonapartist and authoritarian turn raises fears that Tunisia may take a similar path to nearby Egypt: ‘strong man’ rule in the name of stability and national unity, with the country's security forces embedded at the centre of a highly repressive state apparatus.^19^

In Sudan, a large‐scale popular uprising sparked by the lifting of wheat subsidies began in December 2018. After several months of protest and civil disobedience, the demonstrators successfully ousted long‐standing dictator Omar Al Bashir in April 2019. Following this uprising — arguably the most comprehensive social transformation in the region since the events of 2011 — a power‐sharing agreement was reached in August 2019 between the country's military and the Forces of Freedom and Change (FFC), an alliance of social movements and political parties that had been formed in January 2019. This agreement was meant to achieve a transition away from authoritarian rule and the dominance of a handful of Sudanese generals, but it proved to be short‐lived, given the military's entrenched position in the country's political system and its control of all major economic sectors (Sayigh, 2021). Following the onset of the COVID‐19 pandemic, economic and social conditions in Sudan worsened significantly, with the country experiencing widespread food and fuel shortages, a large spike in public debt, and a spiralling inflation rate that hit 163 per cent in 2020 — by far the highest in the Middle East (IMF, 2021a: 8). In the midst of this crisis, a military coup led by a Sudanese general, Abdel Fattah Al‐Burhan, dissolved the power‐sharing agreement between the FFC and the military on 25 October 2021.

Following Al‐Burhan's coup, protests have once again erupted across the country, led by local resistance committees that are directly challenging the political and economic role of the military. Under the slogan of ‘the three Nos’ — no partnership (with the military), no negotiation (with the military), and no legitimacy (to the military) — these mobilizations have been met with mass arrests and the killing of at least 70 protestors by the Sudanese military and security forces (Al Jazeera, 2022). The Sudanese intelligence forces have regained powers of arbitrary detention (Eltair and Abdelaziz, 2021), and the military has demanded the shutdown of internet and telecommunications services in order to prevent the sharing of information about marches and sit‐ins (Osman, 2021). Despite this repression, protests and strikes are ongoing as of January 2022.

Of the three countries surveyed in this section, Sudan is undoubtedly the starkest confirmation of how the pandemic has been accompanied by an increase in the coercive dimensions of state power. In all cases, however, the legacies of the popular protests that emerged before 2020 have not dissipated. State structures have proven incapable of addressing the multiple social crises that intersect with the pandemic, and this has further eroded the already brittle legitimacy of existing political elites. Despite the considerable variation in state‒class relations and the nature of political power across Lebanon, Tunisia and Sudan, new layers of political activists continue to be drawn into protests and other diverse forms of social mobilization. While it is beyond the scope of this article to explore the emerging debates within these movements, it should be stressed that a rich political discussion is also ongoing, with activists reflecting on the challenges posed by the pandemic and the lessons to be learned from previous modes of organization and protest (Bou Khater, 2021; El Kak, 2021; Omer, 2022).

## CONCLUSION

This article has emphasized the importance of situating the impact of COVID‐19 on the Middle East within the long‐standing trajectory of capitalist development in the region. The principal effect of the pandemic has been to further accentuate many of the developmental features of the region that preceded it: extreme levels of social and economic inequality (experienced across divisions of gender, youth, citizenship and so forth), a sharp polarization of regional wealth and power, and a deep disillusionment with the existing political structures. The experience of the last 18 months highlights how the preceding decades of neoliberal restructuring have weakened the capacity of individuals, households and states to face the crisis. Indeed, the effects of the pandemic have primarily been transmitted through channels that were a direct consequence of preceding economic reform (e.g. export‐led agriculture and import‐dependent food systems, labour market deregulation, privatized health care and social support provision). As such, the region's most vulnerable populations have borne the direct brunt of the pandemic — both at the level of public health as well as through its wider economic effects. In this sense, the pandemic needs to be seen as a catalyst for further uneven development — expressed at both the regional and national scales — not simply as a public health crisis in and of itself.

Given this situation, it is likely that IFIs and more powerful states will attempt to wield the current crisis as they did those that followed the 2011 uprisings — utilizing this moment of profound instability as a means of recuperating and re‐embedding market‐led development models. Civil society organizations warn of a new ‘cult of austerity’, with COVID‐19 emergency loans used to further strengthen the power of IFIs through conditionalities and coercive advice (ActionAid, 2021: 9). A large rise in debt levels is predicted for many countries in the region (see Table 2 above), and government expenditure as a proportion of GDP is predicted to fall across the Middle East from 2022 onwards (including in the poorest and most conflict‐affected countries) (Ortiz and Cummins, 2021). ‘Vaccine politics’ will undoubtedly play a major role within this; the wide disparities in financial capacities across the region — as well as control over supply chains and logistics infrastructures — significantly strengthen the power of wealthier states (regionally and internationally) to impose political and economic conditions in return for access to vaccines.

Nonetheless, despite this worsening crisis, the future trajectories of the region's development will continue to be shaped by political struggle. The pandemic hit the Middle East at a moment of resurgence in popular mobilization, encompassing a large number of countries, including those that stood somewhat outside the experience of the 2011 uprisings. While the initial phase of the pandemic saw the roll‐out of emergency restrictions that dampened and de‐mobilized these street protests, it is clear that the core grievances that drove these movements have not disappeared but, rather, deepened. This has precipitated the renewal of protest and political mobilization in several countries across the region. It has also led to a further entrenchment of authoritarianism in the Middle East, expressed through increased repression of political activism and a re‐embedding of the region's militaries and security forces within the state form.

In this manner, the pandemic highlights the importance of thinking about cycles of protest and capitalist crisis through diverse timeframes and temporalities. The foregoing narrative has stressed the *longue durée* of capitalist crisis in the region, which set the stage for (and was accelerated by) the pandemic over the last 18 months. In contrast, moments of rebellion and protest tend to be marked by more non‐linear and discontinuous temporalities that Walter Benjamin contrasted to the kind of homogeneous, empty time that we typically experience (Benjamin, 1940). The pandemic has illustrated how protest movements can recuperate and validate earlier periods of struggle and leave their traces in the present — even when apparently unsuccessful at the level of the state. In this sense, it is significant that the pandemic hit the Middle East at a relatively high point of popular struggle in several countries across the region, and that practices of solidarity have been crucial to survival for many communities faced with the ‘organised abandonment’ (Gilmore, 2008) of the neoliberal state. For these reasons, we should not discount the likelihood of a new wave of political mobilizations coming out of the pandemic.

## Footnotes

1 Given the regional categorization employed in much of the official statistical data drawn upon in this article, we use the term ‘Middle East’ to also encompass the countries of North Africa (Algeria, Egypt, Libya, Morocco and Tunisia). Our principal focus is on the Arabic‐speaking countries of the region and Israel in the context of its occupation of the West Bank and Gaza Strip, although we make mention of Iran and Turkey where relevant.

2 Calculated from data obtained from Worldometer (www.worldometers.info/coronavirus/). Countries included are Algeria, Bahrain, Egypt, Iran, Iraq, Israel, Jordan, Kuwait, Lebanon, Libya, Morocco, Occupied Palestinian Territory, Oman, Qatar, Saudi Arabia, Sudan, Syria, Turkey, Tunisia, United Arab Emirates and Yemen.

3 Positivity rate is the proportion of positive cases relative to the number of tests, with more than 5 per cent indicating uncontrolled infection. Iraq, Jordan, Libya, Morocco, Occupied Palestinian Territory, Oman and Tunisia all experienced positivity rates of 20‒50 per cent in periods over 2020 and 2021 (https://ourworldindata.org/coronavirus‐testing#testing‐for‐covid‐19‐background‐the‐our‐world‐in‐data‐covid‐19‐testing‐dataset). Positivity data are not available for Egypt, Lebanon, Sudan or Yemen.

4 The GCC countries are Bahrain, Kuwait, Oman, Qatar, Saudi Arabia and United Arab Emirates, also referred to as ‘Gulf states’.

5 ILOstat database, figures for MENA 2019; see www.ilostat.ilo.org/data/ (accessed 3 September 2020).

6 For a nuanced, critical discussion of this study and some of the assumptions embedded in its conclusions, see Harris (2017).

7 ILOSTAT database, figures for MENA (www.ilostat.ilo.org/data/; accessed 3 September 2021).

8 In Israel and the GCC states, government expenditure as a share of GDP grew by 7 and 6.7 percentage points respectively in 2020. However, across MENA as a whole, government expenditure increased by only 0.8 percentage points. Algeria, Egypt, Lebanon, Libya, Sudan and Yemen registered a decrease in government expenditure as a share of GDP over 2020 (in the context, moreover, of a decline in overall GDP) (IMF, 2021b).

9 For Saudi Arabia alone, more than 257,000 workers left in the third quarter of 2020. The Indian state of Kerala, a major source of migrants to the Gulf, saw 700,000 migrants return home in 2020 (World Bank, 2021b: 10).

10 The ILO estimates an 11.7 per cent decline in remittances for the Middle East in 2020 (ILO, 2021b: 64). According to the World Bank, the Philippines experienced a 14 per cent drop in remittances from Saudi Arabia in 2020, and India saw a 17 per cent decline in remittances from the UAE (World Bank, 2021b: 10, 31).

11 Equidem (2020) provides a detailed account of these schemes and migrant experiences during the pandemic based on interviews with over 200 migrants in Qatar, Saudi Arabia and the UAE.

12 See Migrant‐rights.org ‘The Emergence of the Vaccine Passport’, 6 July 2021. www.migrant‐rights.org/2021/07/the‐emergence‐of‐the‐vaccine‐passport/ (accessed 10 January 2022).

13 Indeed, many Palestinian workers who displayed COVID‐19 symptoms were returned to the West Bank without testing, and Palestinian workers thus became the primary vector for the transmission of the virus into the West Bank. According to the Palestinian Ministry of Health, 74 per cent of cases in the West Bank during the first wave of the pandemic were Palestinians employed in Israel or those in their immediate surroundings (*Quds Press*, 2020). The first Palestinian fatality was a woman who contracted the virus from her son, an industrial worker in an Israeli settlement.

14 The capabilities of the Bahraini app were demonstrated through its use in a television show, ‘Are You at Home?’, which accessed telephone numbers of random individuals and used the app to check whether they were at home. Initially, enrolment in the TV show was mandatory and automatic. While the Bahraini and Kuwaiti apps stand out with regard to surveillance, other Gulf states have also introduced similar technologies (see Amnesty International, 2020).

15 These are: Jordan (38 per cent), West Bank and Gaza Strip (29 per cent), Lebanon (27 per cent), Egypt (20 per cent), Iraq (14 per cent), Algeria (13 per cent), Libya (12 per cent), Syria (5 per cent), Sudan (3 per cent) and Yemen (1 per cent). All vaccination figures in this paragraph are drawn from Our World in Data COVID‐19 dataset. See https://github.com/owid/covid‐19‐data/tree/master/public/data/vaccinations

16 In this situation, hoarding and smuggling have proliferated — especially the smuggling of subsidized fuel to neighbouring Syria. For a country heavily reliant on generators rather than a functioning electrical grid, the high price and shortages of fuel have left many Lebanese households unable to afford electricity. This electricity crisis has been particularly devastating for hospitals battling the pandemic.

17 In August 2020 then‐Prime Minister Hassan Diab resigned in the wake of popular outrage around the Beirut Port explosion. Diab, however, was forced to continue in a ‘caretaker’ role for another year because no alternative government could be formed. The current prime minister, Najib Mikati, is a billionaire businessperson and politician, reputedly the richest person in Lebanon and named in the Pandora Papers for his extensive offshore wealth.

18 For a fascinating analysis of the intersection between COVID‐19 and the territorialization of governance and sectarianism in Lebanon, see the innovative work of the Beirut Urban Lab, American University of Beirut: https://beiruturbanlab.com/en/Details/614/the‐territories‐of‐the‐covid‐19‐response‐in‐lebanon

19 An important distinction in the Tunisian case is the significance of the country's police forces to autocratic rule (rather than the military, as is the situation in Egypt).
